# *In situ* pulmonary mucus hydration assay using rotational and translational diffusion of gold nanorods with polarization-sensitive optical coherence tomography

**DOI:** 10.1117/1.JBO.29.4.046004

**Published:** 2024-04-30

**Authors:** Kelsey J. Oeler, Richard L. Blackmon, Silvia M. Kreda, Taylor Robinson, Melanie Ghelardini, Brian S. Chapman, Joseph Tracy, David B. Hill, Amy L. Oldenburg

**Affiliations:** a University of North Carolina at Chapel Hill, Department of Biomedical Engineering, Chapel Hill, North Carolina, United States; b Elon University, Department of Engineering, Elon, North Carolina, United States; c University of North Carolina at Chapel Hill, Marsico Lung Institute/Cystic Fibrosis/Pulmonary Research and Treatment Center, Chapel Hill, North Carolina, United States; d University of North Carolina at Chapel Hill, Department of Physics and Astronomy, Chapel Hill, North Carolina, United States; e North Carolina State University, Department of Materials Science and Engineering, Raleigh, North Carolina, United States

**Keywords:** optical coherence tomography, polyethylene oxide, mucus, gold nanorods, particle diffusion

## Abstract

**Significance:**

Assessing the nanostructure of polymer solutions and biofluids is broadly useful for understanding drug delivery and disease progression and for monitoring therapy.

**Aim:**

Our objective is to quantify bronchial mucus solids concentration (wt. %) during hypertonic saline (HTS) treatment *in vitro* via nanostructurally constrained diffusion of gold nanorods (GNRs) monitored by polarization-sensitive optical coherence tomography (PS-OCT).

**Approach:**

Using PS-OCT, we quantified GNR translational ($D_{T}$) and rotational ($D_{R}$) diffusion coefficients within polyethylene oxide solutions (0 to 3 wt. %) and human bronchial epithelial cell (hBEC) mucus (0 to 6.4 wt. %). Interpolation of $D_{T}$ and $D_{R}$ data is used to develop an assay to quantify mucus concentration. The assay is demonstrated on the mucus layer of an air–liquid interface hBEC culture during HTS treatment.

**Results:**

In polymer solutions and mucus, $D_{T}$ and $D_{R}$ monotonically decrease with increasing concentration. $D_{R}$ is more sensitive than $D_{T}$ to changes above 1.5 wt. % of mucus and exhibits less intrasample variability. Mucus on HTS-treated hBEC cultures exhibits dynamic mixing from cilia. A region of hard-packed mucus is revealed by $D_{R}$ measurements.

**Conclusions:**

The extended dynamic range afforded by simultaneous measurement of $D_{T}$ and $D_{R}$ of GNRs using PS-OCT enables resolving concentration of the bronchial mucus layer over a range from healthy to disease in depth and time during HTS treatment *in vitro*.

## 1 Introduction

In biomedical research, it is of great importance to assess the nanostructure of biomaterials as it relates to disease progression and transport. For instance, tissue engineers are particularly interested in characterizing the nanostructure of biomimetic scaffolds, as it provides mechanical cues for cellular behavior and function.^1^ Monitoring alterations in pore size, which occur when the nanostructure of the extracellular matrix (ECM) is remodeled during disease progression or regression,^2^^,^^3^ can aid in tracking treatment efficacy.^4^ Understanding the diffusion of nanoparticles within nanostructures can also lead to new applications for drug delivery. For example, the barrier properties of the biopolymeric meshwork of mucus inform the design of drug delivery systems for the lung epithelium.^5^^–^^7^ Mucus nanostructure is also an important marker of lung health: in pulmonary diseases such as cystic fibrosis (CF) and chronic obstructive pulmonary disease (COPD), mucus becomes significantly dehydrated, causing disruption of the mucociliary clearance system (MCC),^8^^–^^11^ which is the primary defense mechanism responsible for trapping and clearing inhaled pathogens from the respiratory system.^12^^–^^16^ The increase in mucus solids concentration (wt. %) as it becomes dehydrated is directly associated with decrease in nanopore size within the mucus macromolecular meshwork. To counteract the dehydrated mucus’ impairment of the MCC, mucus-thinning therapies such as aerosolized hypertonic saline (HTS) are commonly administered.^17^^,^^18^ Although HTS is a low-cost and easily administered treatment,^18^^,^^19^ its effectiveness in improving MCC remains uncertain due to patient-to-patient variability and conflicting therapeutic outcomes.^18^^,^^20^^,^^21^ Here we propose a method, based on nanoparticle probe diffusion, for measuring mucus concentration *in situ* and potentially *in vivo* that may provide new insights for improving mucus-thinning therapies.

Scanning electron microscopy (SEM), transmission electron microscopy (TEM), and confocal microscopy are commonly used to assess nanopore size due to their high resolution.^22^^–^^26^ However, these techniques require segmentation for pore size measurements, generally require staining and, for electron microscopy, additional sample preparation. In comparison, an emerging technique involves measuring the diffusion rate of nanoparticles added to an aqueous macromolecular medium where they are weakly constrained due to intermittent and nonadherent collisions with the macromolecules.^27^ This technique offers a promising alternative to traditional imaging methods, as it allows for minimally invasive measurement of the nanopore size distribution and subsequent estimation of the concentration of solids without the need for staining or segmentation. Plasmonic gold nanorods (GNRs) are particularly amenable to this technique because their optical resonance provides a large optical signal from particles that are sufficiently small (<100  nm) to access pores within a macromolecular medium that is well above the overlap concentration, as relevant to biomaterials.

Traditionally, the diffusion of nanoparticles is measured using dynamic light scattering (DLS). GNRs have been assessed via DLS and found to have predictable rotational and translational diffusion rates in water and aqueous glycerol solutions,^28^^,^^29^ and diffusion of GNRs or related rod-like particles have been proposed for material characterization of polymer solutions.^30^^,^^31^ However, none of these studies have exploited the plasmonic resonance of GNRs to increase detectability within optically dense materials. Furthermore, an imaging platform such as optical coherence tomography (OCT) is particularly advantageous for performing depth-resolved DLS in biomaterials due to its increased depth penetration and small sample volume requirement.^32^^,^^33^ Our group’s prior efforts have combined these two concepts, demonstrating DLS-OCT at the longitudinal surface plasmon resonance of GNRs to spatially resolve rotational^34^ and translational^27^ diffusion rates. In addition, the rapid speckle fluctuation rates exhibited by GNRs (with autocorrelation decay times typically <10  ms) enable real-time imaging of GNR diffusion via DLS-OCT. We have dubbed this method diffusion sensitive optical coherence tomography (DS-OCT) and have shown how it reveals temporally and spatially resolved changes in the nanostructure of polymer solutions, collagen, extracellular matrices, and well-hydrated *ex vivo* pulmonary mucus.^27^^,^^35^^,^^36^

DS-OCT requires the collection of both co-(HH) and cross-(HV) polarized light scattering from the sample in order to compute both translational ($D_{T}$) and rotational ($D_{R}$) diffusion rates of GNRs. Our previous work in pulmonary mucus focused only on computation of $D_{T}$ as an indirect measure of mucus solids concentration (wt. %). By collecting $D_{T}$ in stationary mucus samples, an interpolation curve relating $D_{T}$ to concentration was exploited to depth- and time-resolve concentration within the mucus layer of an *in vitro* human bronchial epithelial cell (hBEC) model during treatment by hypertonic and isotonic salines.^35^ However, our methods were unable to extend to dehydrated, disease-like mucus concentrations >3.5 wt. %. In this article, for the first time, we address this limitation by monitoring both $D_{T}$ and $D_{R}$ together, showing the interplay of how these two types of diffusion change as a function of sample concentration. This report begins by presenting experiments conducted in polyethylene oxide (PEO) solutions due to their utility in mimicking the meshwork of pulmonary mucus.^37^ We first quantify $D_{T}$ and $D_{R}$ of GNRs in PEO solutions with concentrations ranging from those that weakly to moderately constrain the GNRs. We then extend our experiments to human bronchial epithelial (hBE) mucus, measuring $D_{T}$ and $D_{R}$ over a dynamic range of concentrations from hydrated and deemed “healthy” states (<2 wt. %) to more severely dehydrated “diseased-like” states (up to 6.4 wt. %). Importantly, as we demonstrate, the additional computation of $D_{R}$ allows one to extend the dynamic range of available mucus concentrations to 6.4 wt. %. Finally, using a model developed from the hBE mucus experiment results, we estimate the concentration change over depth and time in air–liquid interface (ALI) hBEC cultures treated with HTS.

## 2 Materials and Methods

### 2.1 Polarization Sensitive OCT System

**Fig. 1:**
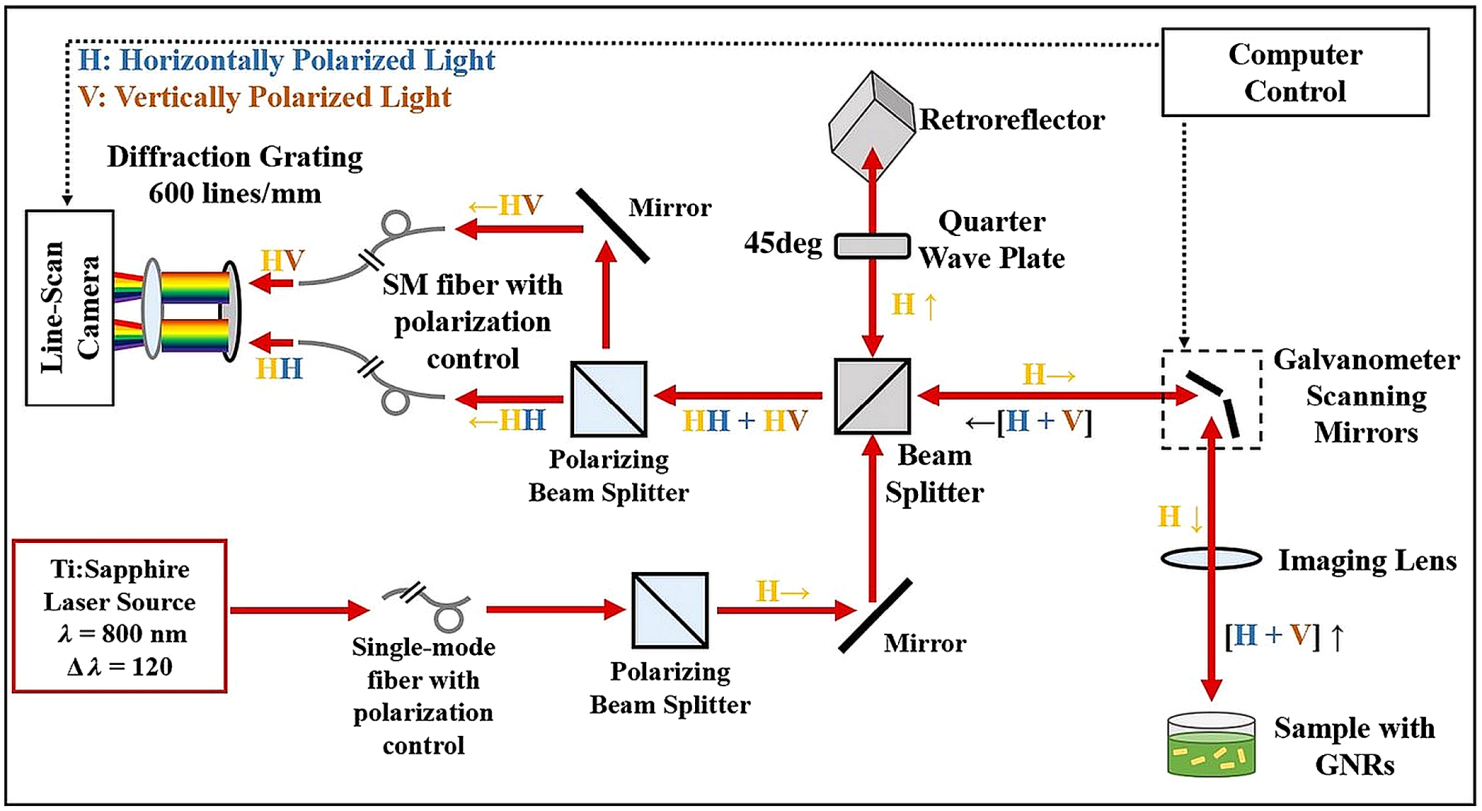
PS-OCT system schematic.

To track both translational and rotational diffusions, a key component of the experimental setup is the polarization sensitive signal collection of the OCT system. A detailed description of our custom polarization sensitive spectral domain OCT system can be found in the previous literature,^34^ but here we provide a brief overview. The system utilizes an 800 nm center wavelength Ti:Sapphire laser (Griffin; KM Labs) with a 120 nm bandwidth as its light source, as shown in Fig. 1. The incident beam on the sample is horizontally polarized, whereas the reference beam as it returns from the retroreflector is linearly polarized at 45 deg (equal parts horizontal and vertical components) after double pass through a quarter wave plate. Light scattered from the sample interferes with the reference beam and the resulting co-polarized (HH: horizontal in and horizontal out) and cross-polarized (HV: horizontal in and vertical out) components are separated by a polarizing beam splitter and directed to a custom spectrometer. The spectral components of both polarization channels are dispersed by a diffraction grating and simultaneously imaged onto each half of a 4096-pixel line scan camera. The OCT system has an axial resolution of 3  μm and a transverse resolution of 12  μm in air, and the power directed on the samples was ∼3.0  mW. The depth of focus is 283  μm. A-line rates were either 25 or 62.5 kHz as indicated in each experiment below.

### 2.2 Diffusion Coefficient Calculations

As a brief review of DS-OCT analysis methods, as previously described in Ref. 27, DS-OCT consists of collecting M-mode OCT images of a GNR-laden sample to depth-resolve (z) translational ($D_{T}$) and rotational ($D_{R}$) diffusion coefficients. M-mode OCT images can then be collected at varying lateral positions (x) or at successive times (t) to construct a cross-sectional (x−z) or dynamic (t−z) image of $D_{T}$ and $D_{R}$; in this study, we perform cross-sectional imaging on stationary samples (PEO, mucus) and dynamic imaging on the mucus layer of *in vitro* ALI hBEC cultures.

For each M-mode OCT image, comprised of A-lines sampled at times $t_{s}$, the co-(HH) and cross-(HV) polarized complex analytic OCT signals, ${\tilde{S}}_{\mathrm{HH}}(t_{s};z)$ and ${\tilde{S}}_{\mathrm{HV}}(t_{s};z)$, respectively, are computed from raw OCT data by standard methods. At each z position, the time averages are subtracted, then the normalized temporal autocorrelations $\left(g_{\mathrm{HH}}(\tau ;z),g_{\mathrm{HV}}(\tau ;z)\right)$ are computed. To suppress noise, the autocorrelations are then averaged in depth windows of 20 pixels or less; each window is considered a region of interest (ROI). By the Siegert relation, these second-order autocorrelations are equated to first-order autocorrelations $g^{\left(1\right)}(\tau ;z)$, which, for GNRs at their longitudinal resonance, are related to $D_{T}$ and $D_{R}$ as follows:^35^

$$g_{\mathrm{HH}}^{\left(1\right)}(\tau ;z)=\frac{5}{9}\;\exp(-q^{2}D_{T}(z)\tau )+\frac{4}{9}\;\exp(-6D_{R}(z)\tau )\exp(-q^{2}D_{T}(z)\tau ),$$ (1)

$$g_{\mathrm{HV}}^{\left(1\right)}(\tau ;z)=\exp(-6D_{R}(z)\tau -q^{2}D_{T}(z)),$$ (2)

where $q=4\pi n/\lambda_{0}$, n is the refractive index of the medium, and $\lambda_{0}$ is the central wavelength of the PS-OCT system. From a linear combination of Eqs. (1) and (2), we calculate the isotropic autocorrelation $g_{\mathrm{ISO}}(\tau ;z)$, which isolates the $D_{T}$ term as follows:

$$g_{\mathrm{ISO}}^{\left(1\right)}(\tau ;z)=\frac{9}{5}g_{\mathrm{HH}}^{\left(1\right)}(\tau ;z)-\frac{4}{5}g_{\mathrm{HV}}^{\left(1\right)}(\tau ;z)=\exp(-q^{2}D_{T}(z)\tau ).$$ (3)

Under our experimental conditions, the 1/e decay time associated with $D_{T}$ is much longer than that associated with $D_{R}$, which simplifies Eq. (2) to isolate the $D_{R}$ term as

$$g_{\mathrm{HV}}^{\left(1\right)}(\tau ;z)\approx \exp(-6D_{R}(z)\tau ).$$ (4)

Decay times $\tau_{\mathrm{ISO}}$ and $\tau_{\mathrm{HV}}$ are extracted by fitting Eq. (3) for $\tau \leq \tau_{1/e}$ and Eq. (4) for $\tau \leq \tau_{1/e^{2}}$, where $\tau_{1/e}$ and $\tau \leq \tau_{1/e^{2}}$ are the delay times at which $g^{\left(1\right)}$ falls below 1/e and $1/e^{2}$, respectively; this excludes noisy tails in the autocorrelations from the analysis. One exception to this rule was PEO solutions with concentrations >1.5%, for which a fit range for Eq. (4) only up to $\tau_{1/e}$ was used. For *in vitro* hBEC cultures, a dynamic $\tau_{\mathrm{ISO}}$ threshold was applied such that a fit range of $\tau \leq \tau_{1/e^{2}}$ was used if fittings had fewer than 4 data points. For all fittings, if fewer than 4 data points were available, the ROI was excluded from further analysis. Also the zero-delay point g(τ=0) was excluded from all fittings to avoid the contribution of shot noise.

Several criteria were used to determine which ROIs were valid for analysis. Criterion 1 is a top surface detection method to exclude ROIs above the sample or too close to the top surface, which exhibits a strong specular reflection. For PEO and stationary mucus samples, valid ROIs start 15 pixels below the top surface detected (1  pixel≈1.5  μm in depth) and 5 pixels below for the hBEC cultures. ROIs are 20 pixels in depth for stationary mucus and 3 pixels in depth for hBEC cultures. Criterion 2 excludes ROIs that are below an average co-polarization (HH) signal threshold above the background noise. Criterion 3 excludes ROIs that do not exhibit valid decay times discussed in Ref. 36. Briefly, we reject ROIs where the decay anisotropy $\tau_{\mathrm{ISO}}/\tau_{\mathrm{HV}}<1.5$, because dynamic scattering from GNRs exhibits higher decay anisotropy. Additionally, $\tau_{\mathrm{ISO}}$ and $\tau_{\mathrm{HV}}$ must lie within the dynamic range of the OCT system (significantly longer than the sampling time and shorter than the total acquisition time). Criterion 4 applies a threshold for autocorrelation fits based on the coefficient of determination ($R^{2}$), for PEO solutions $R^{2}>0.88$ and for stationary mucus $R^{2}>0.90$. Criterion 5 was applied only to PEO and stationary mucus samples, where all diffusion coefficients more than five standard deviations from the mean were assessed visually for air bubbles and other imaging artifacts and manually excluded if appropriate. The fourth and fifth criteria were not applied to *in vitro* hBEC culture studies because mucus hydration was expected to be heterogeneous and dynamically changing.

### 2.3 Gold Nanorods

GNRs stabilized by cetyltrimethylammonium bromide were synthesized following an established protocol.^38^ The GNRs were coated with a 2 kDa molecular weight polyethylene glycol (PEG) methyl ether thiol (Sigma Aldrich) to prevent them from adhering to polymer and mucin macromolecules.^39^ Three batches of GNRs were used in the experiments reported. Transmission electron microscopy (TEM) was performed on each batch to measure the gold core size distributions and PEG coating thicknesses. The mean core sizes were 68×19  nm for batch 1, 70×22  nm for batch 2, and 80×22  nm for batch 3. The thickness of the PEG coating has been measured previously as ∼0.5  nm, which is half of the distance between the sides of nanorods that have dried aligned next to each other on the TEM substrate.^27^ The sizes of the GNRs were chosen to be on the same scale as the nanopores of mucus (∼0.2 to 1  μm^40^). Therefore, their Brownian motion is expected to be hindered by the mucus macromolecules, and corresponding diffusion rates are expected to depend on the mucus concentration. The hydrodynamic radius ($R_{H}$) of the batches, ∼19,22, and 24 nm, respectively, is calculated using GNR length (L) and width (W) according to the following equation:^27^

$$R_{H}=\frac{L}{2(\ln(\frac{L}{W})+0.312+0.565(\frac{W}{L})-0.1{\left(\frac{W}{L}\right)}^{2})}.$$ (5)

### 2.4 Sample Preparations and Experimental Procedures

#### 2.4.1 Polymer samples

PEO of molecular weight ($M_{W}$) 4 MDa was prepared into 5 wt. % stock solutions. To prepare a stock solution, PEO powder was stirred constantly in 80°C distilled water for 1 week. From the stock solution, samples ranging from 0 to 3 wt. % solids were prepared. GNRs (batch 1: 68×19  nm) were added to each sample to a final concentration of 1% solids by volume, which is low enough to avoid significant contributions from particle–particle collisions. Polymer solutions were imaged in polystyrene microwells with the PS-OCT system. For all trials, cross-sectional (x−z) B + M-mode images (3  mm×1.5  mm in x×z in the sample) comprised of 30 M-mode images with 30,000 A-lines each were collected sequentially at a line rate of 62.5 kHz. Samples were imaged sequentially at room temperature.

#### 2.4.2 Mucus samples

The stock mucus samples were harvested from hBEC cultures and diluted to concentrations ranging from 1 to 6.4 wt. % with 1× distilled phosphate buffered saline (DPBS) as previously described.^41^ GNRs were mixed into individual mucus samples to a final concentration of 1% volume fraction. GNR batch 2 (70×22  nm) was used in both trials 1 and 2. Samples were imaged in polystyrene microwells with the PS-OCT system. Samples of each trial were imaged sequentially at room temperature. For each sample, cross-sectional (x−z) B + M-mode images were collected (3  mm×1.5  mm in x×z in the sample) comprised of 30 M-mode images with 30,000 A-lines each, collected sequentially at a line rate of 62.5 kHz. Each sample was imaged in two elevationally displaced (y) locations for greater spatial averaging, therefore comprising a total of 60 lateral sites sampled on each of the 2 samples at each concentration.

#### 2.4.3 ALI cell cultures

Calu-3 cells were cultured under ALI conditions.^42^^,^^43^ After 10 days, when there was adequate lumenal mucus accumulation, cells were maintained at ∼37°C in HEPES-buffered HBSS-based solution (basolateral solution) for imaging. A 10  μL isotonic saline and GNR mixture was premixed to contain ∼1% by volume batch 3 GNRs (80×22  nm) and deposited 6 h before OCT imaging. After initial images were collected, a 10  μL HTS and batch 3 GNR mixture, ∼1% by volume to maintain existing concentration of GNRs in the surface layer, was deposited on top of the cell culture. The cell culture was imaged near the well wall at a 10-to-20-deg angle to avoid imaging the meniscus. 99 total M-mode images comprised of 4000 A-lines were collected at a line rate of 25 kHz all at the same transverse location in the sample. The dynamic (t−z) M-mode images were collected every 3.3 s for 4.5 min, and then every 13.3 s for an additional 4 min.

## 3 Results

### 3.1 Diffusion Coefficients of GNRs in Polyethylene Oxide Solutions

**Fig. 2:**
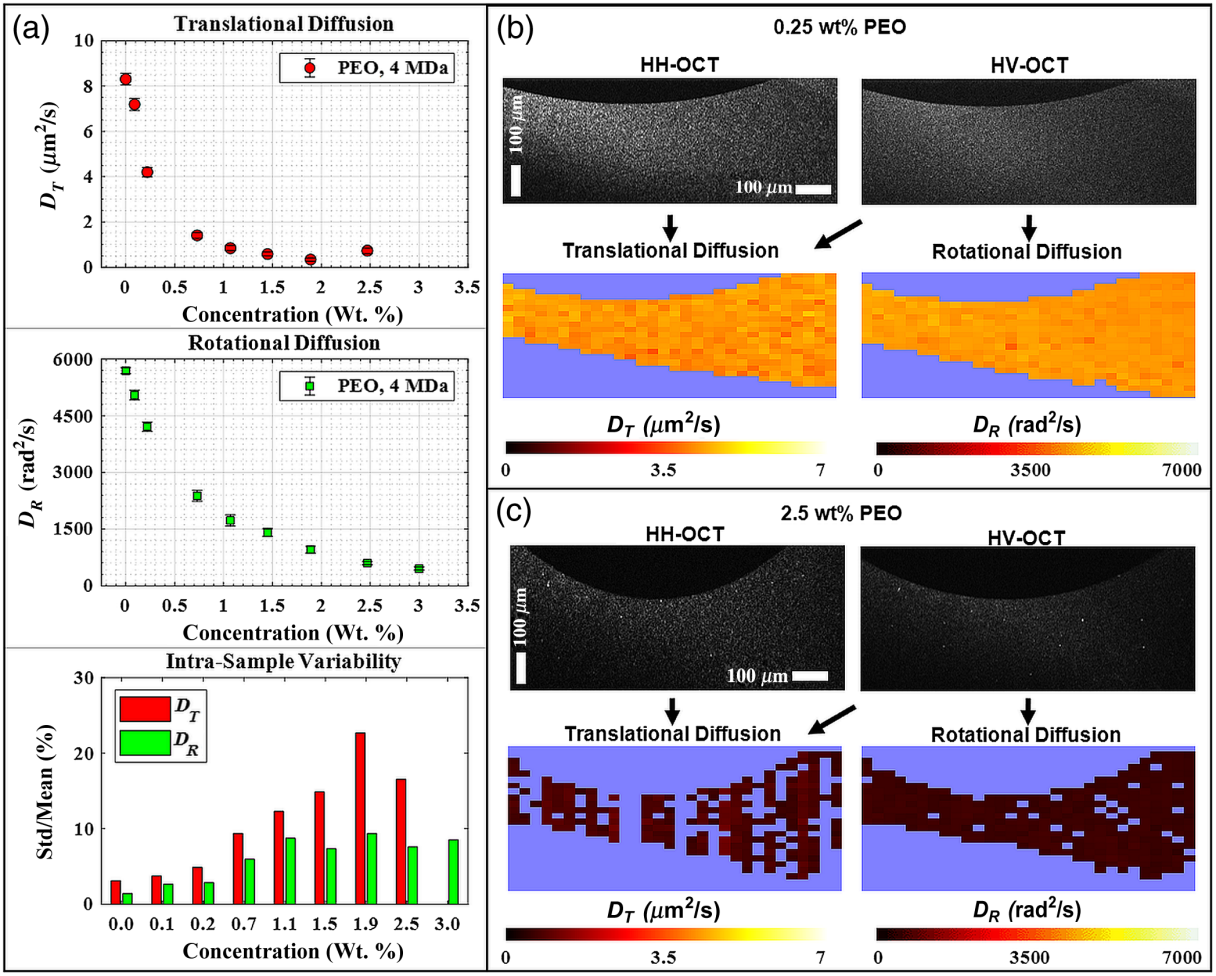
GNR diffusion in aqueous 4 MDa PEO solutions. In the top panels of (a), $D_{T}$ and $D_{R}$ are plotted as a function of PEO solids concentration. In many cases, error bars are not visible due to small standard deviation size. Zero concentration represents GNR diffusion in solvent (distilled water). In the bottom panel, intrasample variability is presented as the percent ratio of the standard deviation to the mean for both $D_{T}$ and $D_{R}$. In panels (b) and (c), co-polarized (HH) and cross-polarized (HV) B-mode images of the 0.25 and 2.5 wt. % PEO solutions are displayed along with corresponding $D_{T}$ and $D_{R}$ values within each ROI derived from B + M-mode images; excluded ROIs based on thresholding criteria described in the methods are displayed as the background color.

The first set of experiments involved measuring GNR diffusion rates in PEO solutions because they are easily accessible, and at Megadalton molecular weights their nanostructure can closely resemble that of mucus.^37^ Figure 2 summarizes these measurements, which were spatially resolved within x−z cross sections of PEO solutions of varying concentrations, then averaged as described above to provide a single value of $D_{T}$ and $D_{R}$ for each concentration. The top panel of Fig. 2(a) illustrates the concentration-dependence of the $D_{T}$ of GNRs in 4 MDa PEO, which exhibits a similar trend as in our previous report using 1 MDa PEO samples with differently sized GNRs:^27^ $D_{T}$ in solvent (distilled water) is $∼8.5\;\mu m^{2}/s$ and decreases rapidly as concentration increases. At concentrations higher than 1 wt. %, $D_{T}$ slows down to $<1\;\mu m^{2}/s$. The middle panel of Fig. 2(a) shows the rotational diffusion rate of GNRs measured within the same samples as translational diffusion. The concentration dependence of $D_{R}$ in 4 MDa PEO exhibits the same trend observed in $D_{T}$ such that diffusion is hindered by increasing concentration. However, in contrast with the $D_{T}$ results, the decay of $D_{R}$ with concentration is less rapid, and $D_{R}$ continues to decrease across all higher concentrations. $D_{R}$ is $>5000\;{\mathrm{rad}}^{2}/s$ in solvent and is reduced by a factor of 10 at our highest sample concentration reported (3 wt. %). As shown in the lower panel of Fig. 2(a), in lower concentration samples (<1 wt. %), the variability (or precision) of $D_{T}$ and $D_{R}$ is <10%. The intrasample variability is determined by dividing the standard deviation by the mean and expressing the result as a percentage, which is often referred to as the coefficient of variation. At higher concentrations, the variability of the $D_{T}$ measurements increases well over 10%, whereas $D_{R}$ measurements maintain low variability across all samples. Figures 2(b) and 2(c) display examples of spatially resolved diffusion measurements at low and high concentrations of PEO. Both $D_{T}$ and $D_{R}$ diffusion maps appear to be homogeneous with no spatially varying noise, which is consistent with PEO samples being well-mixed. However, in the higher concentration sample of Fig. 2(c), $D_{R}$ is defined in nearly twice the number of ROIs defined by $D_{T}$.

The magnitude of the reduction in GNR diffusion in PEO solutions compared to solvent depends on the degree to which the GNRs are confined by the PEO meshwork. The relative degree of confinement may be thought of in terms the size of the hydrodynamic radius of the GNRs, $R_{H}$, compared to the correlation length of the polymer solution ξ. In the prior work, we defined a weakly constrained regime as one where $D_{T}$ was reduced by less than a factor of 10 from that of solvent and found that this occurred when $R_{H}/\xi <2.2$ in 1 MDa PEO Ref. 27. Experiments in 4 MDa PEO, we observe a reduction in $D_{T}$ by a factor of 10 at PEO concentrations ≥1.1 wt. %, which, given an $R_{H}$ of 19 nm for this nanorods batch and ξ of 14 nm for 4 MDa PEO at 1 wt. %, corresponds to a weakly constrained regime defined by $R_{H}/\xi <1.4$. For concentrations above 1.1 wt. %, which we will consider to be strongly constrained, $D_{T}$ was no longer monotonically decreasing and could not be extracted at the highest concentration of 3.0 wt. %. In comparison, $D_{R}$ continued to monotonically decrease for increasing concentration in the strongly constrained regime, which effectively extends the dynamic range of measurable PEO concentrations to 3.0 wt. % and possibly further. Added support for the ability to extend the dynamic range is seen by assessing the intrasample variability, where fine $D_{T}$ and $D_{R}$ precision is evident in the weakly constrained regime, and only $D_{R}$ precision is fine in the strongly constrained regime.

There are several potential explanations for the varying responses of $D_{T}$ and $D_{R}$ versus PEO concentration. First, $D_{T}$ measurements tended to exhibit lower $R^{2}$ values from autocorrelation fittings; this caused many ROIs to be rejected via criterion 4 above where the $R^{2}$ threshold was applied, as observed in Fig. 2(c) at 2.5 wt. %; it is also the reason $D_{T}$ could not be extracted at the highest concentration measured (3.0 wt. %). We believe that the lower $R^{2}$ for $D_{T}$ is either due to the longer $\tau_{\mathrm{ISO}}$ values at high concentrations, which become a significant fraction of the measurement time, causing noise artifacts in the autocorrelation traces, or possibly because the strongly constrained GNR motion no longer fits a model that can be described by simple diffusion. In comparison, $D_{R}$ depends only upon $\tau_{\mathrm{HV}}$, which is always shorter than $\tau_{\mathrm{ISO}}$ for the GNRs in our experiments due to decay anisotropy;^36^ as GNR confinement increases and decay times increase, the smaller $\tau_{\mathrm{HV}}$ better remains within the dynamic range of the measurement compared to $\tau_{\mathrm{ISO}}$. The fact that $D_{R}$ is more easily measurable may also suggest a fundamental difference in how diffusive prolate particles rotate versus translate during strong confinement in polymeric solutions, an area of active research.^44^

### 3.2 Diffusion Coefficients of GNRs in Stationary Pulmonary Mucus

In our previous publications,^27^^,^^35^ we reported that $D_{T}$ is sensitive to hBE mucus concentrations ≤2.5 wt. %, both stationary and on actively transporting hBE cultures (*in vitro*). Mucus ≤2 wt. % is considered well-hydrated and would be expected during a state of “healthy” pulmonary mucus production. To extend the capability of DS-OCT to measure the lower mucus hydration levels associated with pulmonary disease-like states, we must perform diffusion experiments on more heavily dehydrated mucus samples (>3 wt. %). Here we expand our investigation of GNR diffusion to a broader range of mucus concentration, up to 6.4 wt. %, and report values of $D_{R}$ in hBE mucus for the first time.

**Fig. 3:**
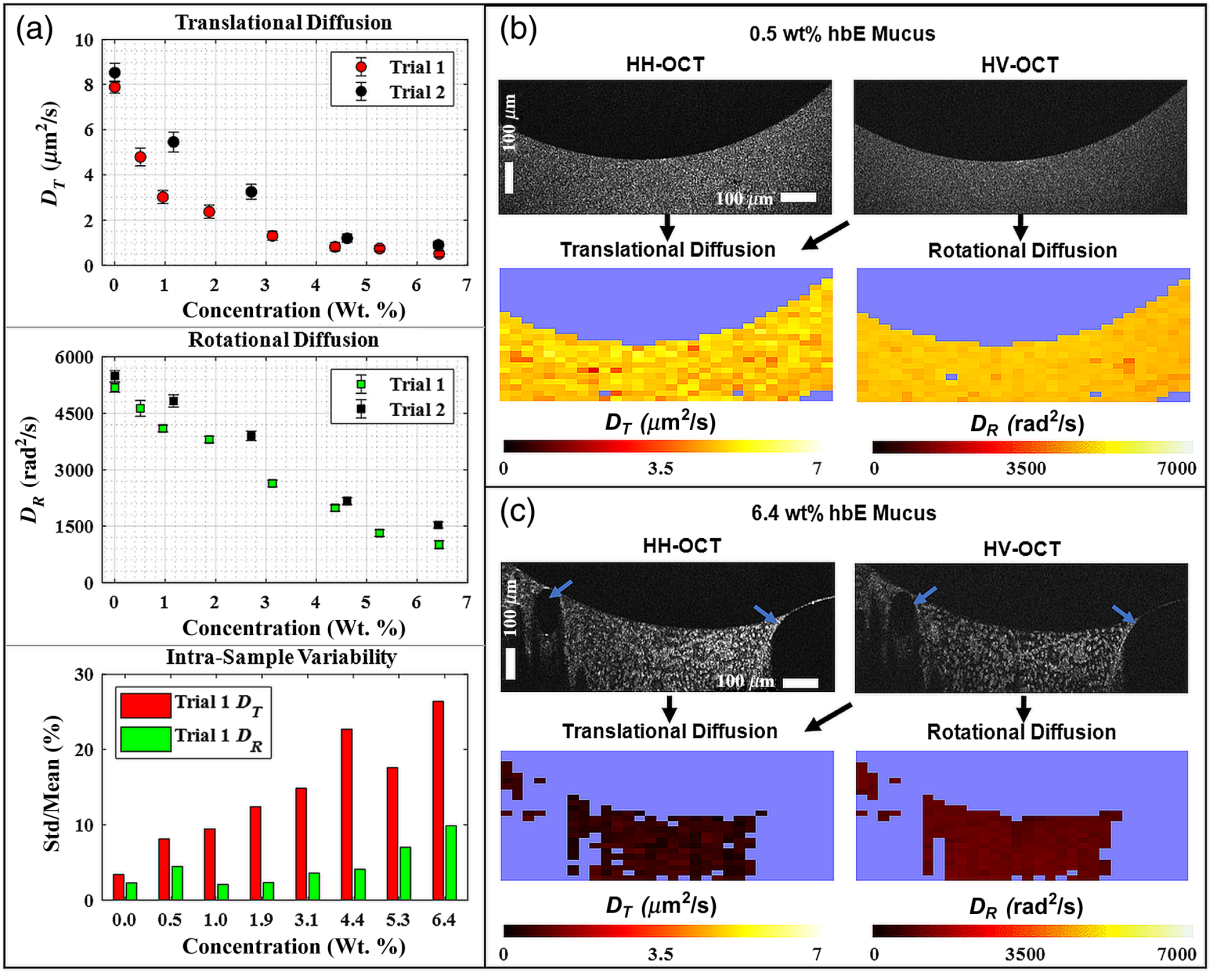
GNR diffusion in hBE mucus sample. In the top panels of (a), $D_{T}$ and $D_{R}$ are plotted as a function of mucus solids concentration. In many cases, error bars are not visible due to small standard deviation size. Zero concentration represents GNR diffusion in solvent (DPBS). In the bottom panel, intrasample variability is presented as the percent ratio of the standard deviation to the mean for both $D_{T}$ and $D_{R}$. (b), (c) Co-polarized (HH) and cross-polarized (HV) B-mode images of the 0.5 and 6.4 wt. % hBE samples are displayed along with corresponding $D_{T}$ and $D_{R}$ values within each ROI derived from B + M-mode images; excluded ROIs based on thresholding criteria described in the methods are displayed as the background color. (c) Multiple ROIs are rejected due to air bubbles (indicated by blue arrows) in the 6.4 wt. % sample.

As shown in Fig. 3(a), while trial 2 tended to exhibit higher $D_{T}$ and $D_{R}$ values at all mucus concentrations relative to trial 1, similar concentration-dependent trends were observed overall. In mucus samples below 2 wt. %, $D_{T}$ quickly decreases with respect to concentration while remaining above $2\;\mu m^{2}/s$. For GNRs in dehydrated mucus (>2 wt. %), we observe a general trend that $D_{T}$ slows at smaller increments with increasing concentration and plateaus at $∼0.5\;\mu m^{2}/s$ [Fig. 3(a)]. In well-hydrated samples, $D_{R}$ retains a significant fraction of its value in solvent, above $∼3000\;{\mathrm{rad}}^{2}/s$, then gradually decreases with further increases in mucus sample concentration. At the highest reported concentration, $D_{R}$ is calculated to be ∼1000 and $∼1500\;{\mathrm{rad}}^{2}/s$ in trials 1 and 2, respectively. Across all reported sample concentrations, outside of the solvent data, the variance in $D_{R}$ is less than half of that observed in $D_{T}$. $D_{T}$ intrasample variability is over 10% in all reported concentrations over 1 wt. %, whereas $D_{R}$ variability remains under 10%.

Overall, the trends in $D_{T}$ and $D_{R}$ versus mucus concentration closely mirror those observed in PEO solutions. If we define, as for the PEO experiments above, a weakly constrained regime of GNR diffusion as that when $D_{T}$ is ≥1/10 the value in solvent, GNRs transition to being strongly constrained when mucus concentration exceeds ∼4.5 wt. %. However, $D_{R}$ continues to be sensitive to mucus concentration in this strongly constrained regime. Also, similar to results in PEO, diffusion colormaps of the highest concentration sample in Fig. 3(c) show that there are multiple ROIs where $D_{R}$ is defined while $D_{T}$ is not (criterion 4). We note that samples in trials 1 and 2 were mixed under slightly different temperatures, potentially contributing to the observed differences in $D_{T}$ and $D_{R}$. The higher concentration samples are prone to air bubbles during the mixing process, an example of which can be seen in Fig. 3(c). Large areas of rejected ROIs are due to air bubbles.

Although $D_{T}$ measurements are a valid tool for assessing the nanostructure of well-hydrated mucus samples, these results with mucus point to $D_{R}$ being more sensitive to the concentration of dehydrated mucus samples. Incorporating our previously published translational diffusion in hBE mucus results in Ref. 27 and the above reported hBE experiments, we can define an assay for estimating the concentration of mucus given diffusion coefficient measurements. We first normalized all diffusion coefficients with respect to the diffusion coefficient in solvent measured on the same day, $D_{\text{norm}}=D/D_{\text{solvent}}$. A linear regression was then taken of $D_{T,\text{norm}}$ versus concentration over a well-hydrated range (0 to 2 wt. %), resulting in

$$\mathrm{wt.}\%=-2.75D_{T_{\text{norm}}}+2.53,$$ (6)

with $R^{2}=0.86$. Similarly, a linear regression of $D_{R,\text{norm}}$ over all concentrations (0 to 6.4 wt. %) was found to be

$$\mathrm{wt.}\%=-8.62D_{R_{\text{norm}}}+8.14,$$ (7)

with $R^{2}=0.95$. Further details on this regression may be found in the Supplementary Material.

To apply this assay, we collect $D_{T}$ and $D_{R}$ measurements in a mucus sample and solvent (saline), then compute weight percents from Eqs. (6) and (7). If the concentration falls between −0.5% and 2% for $D_{T}$, or between 1% and 8% for $D_{R}$ (extrapolating somewhat from the maximum measured value of 6.4 wt. %), we consider it valid and exclude values that fall outside these ranges. In the case where we have valid concentration values from both $D_{T}$ and $D_{R}$, we take the average. One limitation of this method is the potential variation in $R_{H}$ values among different batches of GNRs. To help account for potential nanorod size variability in $D_{T}$ measurements, we incorporated three GNR batches with distinct sizes when calculating the $D_{T}$ trendlines. The GNR batch used in this experiment has a size of 70×22  nm with a corresponding $R_{H}$ of 22 nm. The additional two batches of GNRs incorporated from our previous publication,^27^ sized 83×22  nm and 62×10  nm, had corresponding $R_{H}$ of 24 and 19 nm.

### 3.3 Diffusion Coefficients of GNRs on In Vitro Mucus Under HTS Treatment

**Fig. 4:**
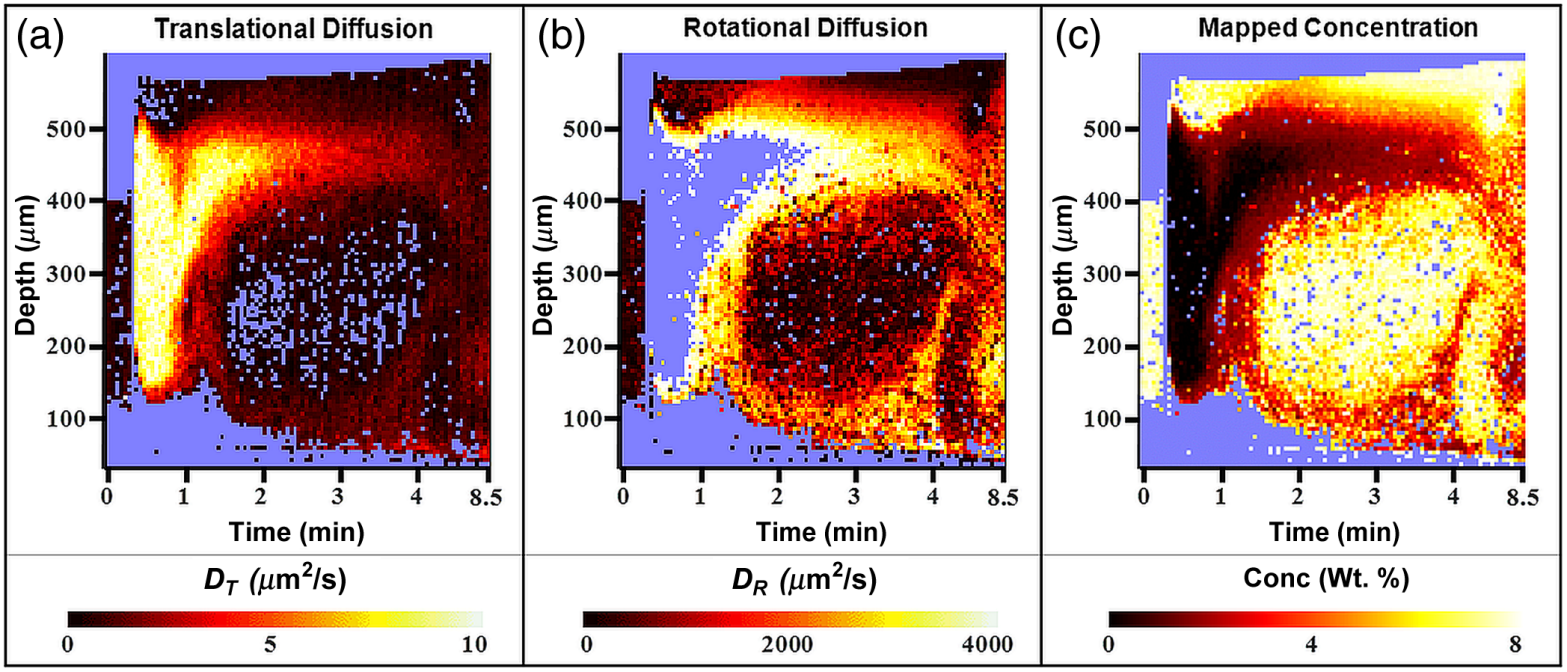
(a), (b) $D_{T}$ and $D_{R}$ in ALI cultures with HTS introduced are color mapped. (c) The diffusion coefficients are mapped to mucus concentration using trendlines produced from stationary mucus experiments. If an ROI had a valid measurement of both $D_{T}$ and $D_{R}$, the calculated concentrations were averaged for a final reported value.

To mimic the airway epithelium physiology, Calu-3 cells were cultured under ALI conditions. The cell cultures were imaged with PS-OCT immediately before and over the course of 8.5 min after treatment with HTS. The mucus layer, which already contained GNRs introduced 6 h prior, was ∼300  μm thick before saline introduction. After topically introducing HTS with additional GNRs, the layer was ∼500  μm thick. We note that the bottom surface positions shift after the addition of the saline due to the refractive index of the liquid causing added optical delay in OCT. In Fig. 4(a), we observe $D_{T}$ of GNRs in the mucus layer of the ALI culture over depth and time. We define a depth of zero as the top of the epithelium where mucus is secreted from mucus goblets. Initially $D_{T}$ is rapid, with rates much higher than $7\;\mu m^{2}/s$ during HTS introduction. After 1 min, $D_{T}$ is measurable in ROIs close to the ALI but exhibits an increasing number of invalid ROIs in regions below, closer to the epithelium, likely due to high concentration that is outside the measurement range for $D_{T}$. Interestingly, at depths closer to the epithelium, $D_{T}$ starts to increase after 3 min, whereas it decreases near the top surface, resulting in a more homogenized value of $D_{T}$ over depth at 8.5 min.

Figure 4(b) reveals the corresponding $D_{R}$ of GNRs in the mucus layer of the ALI culture. Over the first minute, $D_{R}$ is largely undefined in between the ALI and the epithelium. The regions of interest that are quantified have high $D_{R}$, $>3500\;{\mathrm{rad}}^{2}/s$. Beyond 2 min, $D_{R}$ in ROIs close to the surface remain consistently high. Underneath this area, a tall mass of high concentration mucus is revealed by $D_{R}$ measurements, in which the diffusion is hindered more than those at the ALI. Comparing Figs. 4(a) and 4(b), we see the complementary nature of $D_{T}$ and $D_{R}$, with $D_{T}$ having fewer invalid ROIs in regions of lower concentration and $D_{R}$ accessing higher concentration regions; at the same time, the overlapping ROIs of the two appear to follow the same depth- and time-resolved trends.

Using the trendlines defined in Eqs. (6) and (7) for relating $D_{T}$ and $D_{R}$ measurements to mucus concentration, in Fig. 4(c), we mapped the concentration of the mucus within the ALI cell culture in depth and time. By employing the new calibration method that exploits the complementary nature of the two diffusion coefficients, we produce a more spatially and temporally contiguous plot. Before HTS is introduced (at time periods <∼20  s), mucus appears hard-packed within a 400  μm layer. Immediately after HTS is introduced, we observe a ∼500  μm layer of liquid that appears to be mostly solvent, then a thin, highly concentrated layer of mucus near the ALI. Over time, high concentration mucus also appears lower down near the epithelium, and the depth-dependent concentration is generally heterogeneous. Between 4 and 8.5 min, the mucus appears to continue to mix and homogenize, likely as a result of ciliary activity on the epithelium, which we have found is more active in response to HTS than to isotonic saline.^35^ Bearing in mind that “healthy” mucus is considered to be ∼2 wt. %, we see that the mucus layer is nearing but has not quite reached, this value by the end of the experiment. In our previous publication,^35^ we observed the reinitiation of the MCC in time-lapse OCT images during HTS treatment on hBEC. These results suggest the promising advantages of incorporating a combination of our advanced DS-OCT-based assay with regular OCT imaging for a comprehensive evaluation of the HTS treatment process in future applications.

## 4 Discussion

The MCC is the respiratory system’s main defense mechanism to inhaled pathogens. Pulmonary diseases, such as CF and COPD, cause mucus to become severely dehydrated and leaves individuals prone to infection. HTS is commonly used to hydrate the mucus to promote better function of the MCC. In this article, we present, for the first time, a method of using translational and rotational diffusion rates of GNRs introduced into the HTS to develop an *in situ* assay of mucus hydration levels. Because mucus is a limited resource, we first quantified diffusion rates in PEO solutions. The rotational diffusion was less hindered than translational diffusion in strongly confining PEO concentrations. A similar effect was observed in stationary hBE mucus samples.

To properly characterize the heterogeneity of a polymeric fluid, a probe must be smaller than the mesh size, or correlation length, of the material but not so small that the probe will never interact with the polymer mesh, i.e., probes must be in a mesoscopic length scale.^45^ As a polymeric material, the mesh size of mucus is defined by the concentration and size of the high molecular weight mucin glycoproteins.^46^ Published values for the mesh size of mucus between healthy-like concentrations and those associated with airway diseases range between 100 nm and 1  μm.^47^ We therefore feel that the 70×22  nm probe size employed by our studies is appropriate to probe the heterogeneity from the nanothrough microscopic length scales. From the measurements in stationary mucus samples, we were able to fit a model to calculate percent weight concentration given $D_{T}$ and $D_{R}$. Applying this model to an hBEC cell culture treated with HTS, we demonstrated that rotational diffusion could provide information of mucus hydration levels up to 6.4 wt. % and may estimate even more highly concentrated mucus as evidenced by extrapolating the regression line out to 8.0 wt. %. The extended mucus concentration range afforded by this method will enable future insights into the heterogeneity and treatment of disease-like mucus.

Regarding potential *in vivo* use, it is noteworthy that appropriately functionalized GNRs can be biocompatible.^48^ GNRs have the potential to be nebulized and administered to the lungs via inhalation. Upon delivery, we would expect them to disperse throughout a highly concentrated mucus layer within minutes. Importantly, GNRs are not expected to penetrate the lung epithelium based upon their size and the exclusion properties of the airway barried^41^ and should eventually be cleared by the MCC. However, additional motion stabilization on the millisecond time scale may be needed in order to accurately capture diffusion rates *in vivo*. We note that related efforts to capture ciliary beating with OCT on similar time scales have already been accomplished.^49^ Mucociliary transport itself is not expected to significantly impact measurements (motion on the order of 40 to 100  μm/s would be expected to decorrelate a coherence volume in ≥0.1  s in our system, which is long compared to the GNR diffusion-associated decay times).^50^

Future applications of measuring both translational and rotational diffusion of GNRs can lie in vast biomedical research areas as biopolymeric meshes are ubiquitous. Overall changes in nanopore size or anisotropy are related to a host of medically relevant processes and diseases. In particular, anisotropic nanostructure may arise from cilia inducing shear on the macromolecular meshwork of mucus or from ECM-altering cells during ECM remodeling processes. For this purpose, we have previously curated a new technique called diffusion tensor OCT (DT-OCT) to determine the anisotropy of a macromolecular pore size using our DS-OCT methods.^51^ We also note that, based on these results, the use of a simplified HV-OCT system (in comparison to the HH + HV-OCT system employed here) to measure rotational GNR diffusion alone may prove sufficient to quantify the higher concentrations of the samples investigated in this work. $D_{R}$ measurements generally exhibited lower intrasample variability (precision), although this does not necessarily imply greater accuracy. At lower concentrations, we found that the HV decay time became too short to be accurately captured by the OCT system’s line rate of 62.5 kHz. Further increasing, the line rate would reduce the SNR and ability to detect GNR diffusion. The exclusive reliance on $D_{R}$ measurements may inadvertently lead to the oversight of low concentration data, and it appears worthwhile to consider the complementary roles of translational diffusion, which is more accurately measured in lower concentration samples, integrated with rotational diffusion for higher concentrations.

## 5 Conclusions

In summary, quantifying the rotational diffusion of GNRs in conjunction with translational diffusion can provide reasonable estimates of mucus hydration levels, applicable to therapeutic treatment monitoring. By incorporating rotational diffusion measurements, the dynamic range of sample concentration expands to include dehydrated mucus concentrations. This enhanced sensitivity allowed for the development of a real-time assay to study mucus hydration *in situ*.

## Supplementary Material



## Data Availability

Code and data can be made available upon request to the corresponding author.
