# Supplementary material for: In situ pulmonary mucus hydration assay using rotational and translational diffusion of gold nanorods with polarization-sensitive optical coherence tomography
Source: J Biomed Opt. 2024 Apr 30;29(4):046004. doi: 10.1117/1.JBO.29.4.046004 (PMC11060333; doi:10.1117/1.JBO.29.4.046004)
Supplement: Supplementary file 1 [file JBO_029_046004_SD001.pdf]

Supplementary Material

In situ pulmonary mucus hydration assay using rotational and translational diffusion of gold nanorods with PS-OCT

Kelsey J. Oeler[1], Richard L. Blackmon[2], Silvia M. Kreda[4], Taylor Robinson[1], Melanie Ghelardini[3], Brian S. Chapman,[3] Joseph Tracy[3], David B. Hill[1,4], Amy L. Oldenburg[1,5,*]

[1]*Department of Biomedical Engineering, University of North Carolina at Chapel Hill, Chapel Hill, NC 27599, USA*
[2]*Department of Engineering, Elon University, Elon, NC 27244, USA*
[3]*Department Materials Science and Engineering, North Carolina State University, Raleigh, NC 27606, USA*
[4]*Marsico Lung Institute/Cystic Fibrosis/Pulmonary Research and Treatment Center, University of North Carolina at Chapel Hill, Chapel Hill, NC 27599, USA*
[5]*Department of Physics and Astronomy, University of North Carolina at Chapel Hill, Chapel Hill, NC 27599, USA*

In this supplemental document, we provide context on the linear regressions used to determine trendlines 3.2. Additionally, we provide visuals for the statistical significance of results presented in Figure 2 and Figure 3 of the main text.

1 Linear Regression for trendline determination

Linear regression analysis was applied to the translational diffusions in mucus trial 1 samples, trial 2 samples, and our previously published translational diffusion findings in stationary mucus [1]. This approach aimed to extract trendlines that could establish a correlation between translational diffusion dependence and the prediction of mucus concentration. Considering the absence of an existing model for this specific relationship, we determined that a linear regression could effectively characterize the range from 0 to 2 wt%. In contrast, for rotational diffusion, the model was deemed suitable for concentrations up to 6.5 wt%.

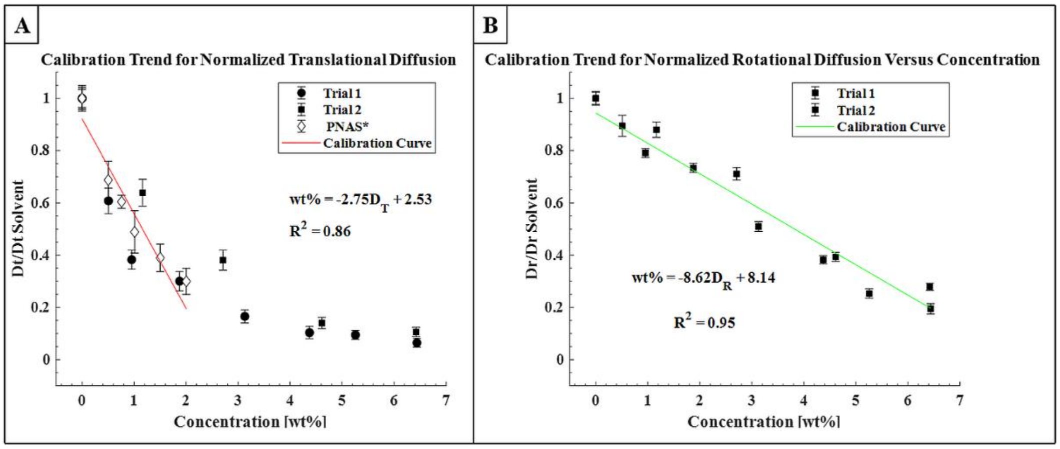

Supplemental Figure 1: (A) Translational diffusion coefficients, normalized by translational diffusion in solvent, are plotted versus mucus concentration. A linear regression is performed over 0 – 2 wt%. *[1] (B) Rotational diffusion coefficients, normalized by translational diffusion in solvent, are plotted versus mucus concentration. A linear regression is performed over 0 – 6.5 wt%.

2 Statistical significances between sample concentration diffusion rates

In assessing the statistical significance across all concentration samples, we employed a one-way ANOVA test. The results revealed a notable distinction between both the translational and

rotational diffusion coefficients, supported by nearly negligible p-values. Supplemental Figure 2 showcases the distinction of each concentration for both translational diffusion and rotational diffusion.

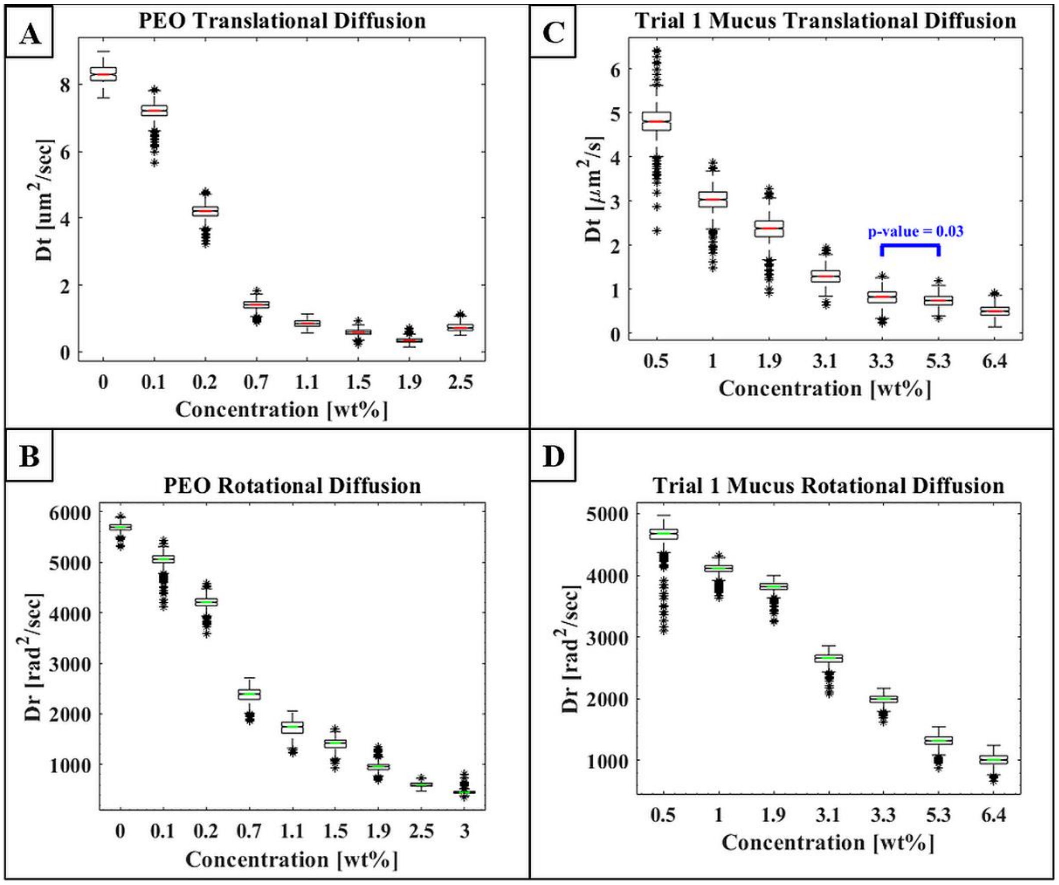

Supplemental Figure 2:Box and whisker plots for PEO samples' translational (A) and rotational (B) diffusion coefficients over a range of concentrations. Box and whisker plots for mucus trial 1 samples' translational (C) and rotational (D) diffusion coefficients over a range of concentrations. The only neighboring sample comparison with a p-value above 0.001 is marked in (C). Red and green lines show the median diffusion coefficient.

References

[1] R. K. Chhetri et al., "Probing biological nanotopology via diffusion of weakly constrained plasmonic nanorods with optical coherence tomography," Proceedings of the National Academy of Sciences of the United States of America 111(41), E4289–E4297, National Academy of Sciences (2014) [doi:10.1073/pnas.1409321111].
